# Acrylic Planas Direct Tracks for Anterior Crossbite Correction in Primary Dentition

**DOI:** 10.5005/jp-journals-10005-1473

**Published:** 2017-02-27

**Authors:** Ashwin Devasya, Naveen K Ramagoni, Mahantesh Taranath, Kamavaram EV Prasad, Mythri Sarpangala

**Affiliations:** 1 Senior Lecturer, Department of Pedodontics and Preventive Dentistry, Kannur Dental College, Kannur, Kerala, India; 2 Professor and Head, Department of Pedodontics, Navodaya Dental College, Raichur Karnataka, India; 3 Professor, Department of Pedodontics, P.M. Nadagouda Memorial Dental College and Hospital, Bagalkot, Karnataka, India; 4 Professor and Head, Department of Pedodontics, Triveni Institute of Dental Sciences Hospital & Research Centre, Bilaspur, Chhattisgarh, India; 5 Reader, Department of Periodontics, Kannur Dental College, Kannur Kerala, India

**Keywords:** Acrylic resin, Crossbite, Malocclusion, Planas direct tracks.

## Abstract

Anterior crossbite is one of the most common forms of malocclusion in sagittal relationship of maxilla and mandible. If not corrected at the earliest, it will cause restriction of normal growth and development of both the jaws. The incidence of anterior crossbite is 4 to 5% in primary dentition. Self-correction may occur at the transient dentition or permanent dentition stage, but treating it should be the first priority. Using either removable or fixed appliances is recommended for the correction, but it depends on the patient cooperation, treatment duration, and parent approval. In this case report, we have used planas direct tracks (PDTs) which helps the forward development of mandible and corrects the malocclusion. With two modifications to PDTs, one is using acrylic instead of composite. It is advantageous to both clinicians and parents by correcting the crossbite efficiently in short duration while taking less chair-side time for fabrication and being economical.

**How to cite this article:** Devasya A, Ramagoni NK, Taranath M, Prasad KEV, Sarpangala M. Acrylic Planas Direct Tracks for Anterior Crossbite Correction in Primary Dentition. Int J Clin Pediatr Dent 2017;10(4):399-403.

## INTRODUCTION

An abnormal relationship between the maxillary and mandibular teeth when the arches are in centric relation is called a crossbite. The crossbite can be unilateral or bilateral and of dental or skeletal origin. The most common is the dental crossbite, involving one or more teeth, along with the adaptation of the soft tissues with the interference, whereas in skeletal, there is alteration of the bone development which causes the asymmetric growth leading to crossbite.^1^

With an occurrence of approximately 4 to 5% of the population in the primary or transitional dentition, anterior crossbite is a common malocclusion that may be diagnosed in growing patients.^2,3^ It may self-correct by transition from deciduous to eruption of incisors. If self-correction does not occur, the anteroposterior relationship of both the jaws becomes worse.^4^ The etiology for this malocclusion is may be combination of skeletal and neuromuscular factors.^5^

The possible treatments may be use of removable and fixed appliances like Hawley appliance with Z spring, Quad helix, and Chin cap. The disadvantages of these being bulky uncomfortable and mainly unable to get patient compliance. Also usage of such appliances for longer period suppresses the mandibles’ anteroinferior growth.^6^ If left untreated, there will be chances of severe establishment of skeletal class 3 malocclusion.

One of the methods of correcting crossbite in primary dentition is by using PDTs by Pedro Planas of Spain in the year 1971.^7^ According to Planas, “Crossbites are very easy to correct, whenever diagnosed early. If not treated, they can produce severe difficulties in the future, due to skeletal modifications that may occur and might be irreversible.” Planas direct tracks were first adapted by Simoes for the correction of anterior and posterior crossbite.^8^

Simoes stated that PDTs should be used in the deciduous dentition only, which cover the occlusal surfaces of the molars, resulting in a flat posterior occlusion until the molars are exfoliated.^9^

They are prism-shaped composite inclined planes either built in laboratory on study models or directly on the tooth. The PDTs are designed such that the distal incline of the upper block occlude with the mesial incline of the lower block such that the mandible will have a posterior path of closure with condyles in centric relation.^8^

The PDTs are small, comfortable, easy to fabricate, and economical. The purpose of this case report is to describe modifications to the PDTs which can be done in a single sitting which will have patient compliance, economical, and correct crossbites in short duration.

**Fig. 1:**
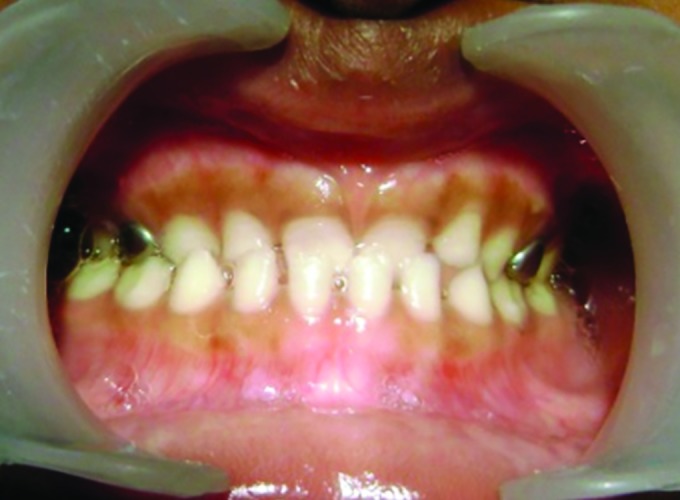
Pretreatment with anterior crossbite

**Fig. 2:**
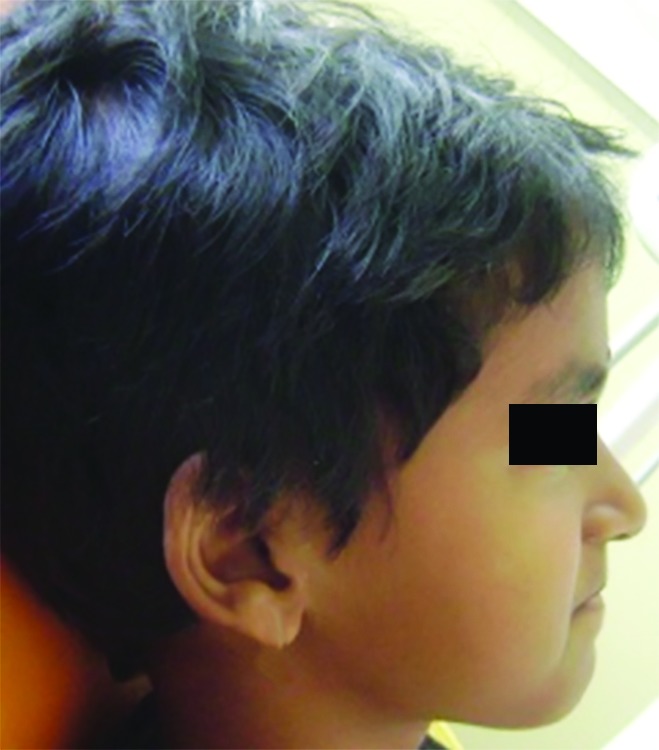
Lateral profile

## CASE REPORT

A mother of a 5-year-old girl came to our department with a chief complaint of small-sized tooth of her child with spaces in between them. After basic clinical examination, it was informed to her and assured that the tooth size is normal to her age, flush terminal molar relation, but the more important problem is the presence of anterior crossbite, which will affect the normal growth and development of the jaws of her child.

After counseling the parents for treatment, on detailed examination it was found that there was complete anterior segmental dental crossbite (Fig. 1). Child had a slightly concave lateral profile (Fig. 2). As a treatment procedure for the same, based on the age and dentition of the child, treatment options like myofunctional appliances and both fixed and removable were explained, and models and photographs showed to parents. Later PDTs were considered because it can only be used in deciduous dentition and other therapies may not be feasible thinking on the child’s cooperation. Ethical clearance and consent of the parents was taken for the treatment.

**Fig. 3:**
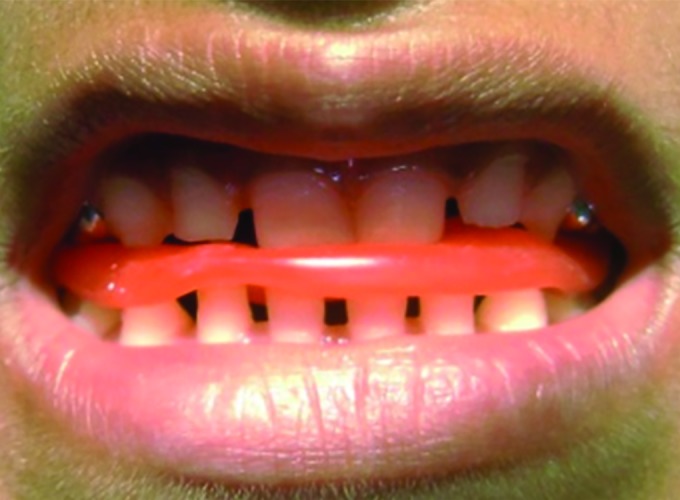
Bite registration

A primary impression was made and working models were prepared. Bite registration was done by making the child to bite in the most posterior position of the mandible that gave edge-to-edge relationship of maxilla and mandible (Fig. 3). Bite was transferred to the working models and mounted on a three-point articulator.

## MODIFICATIONS TO PDTs

**Fig. 4:**
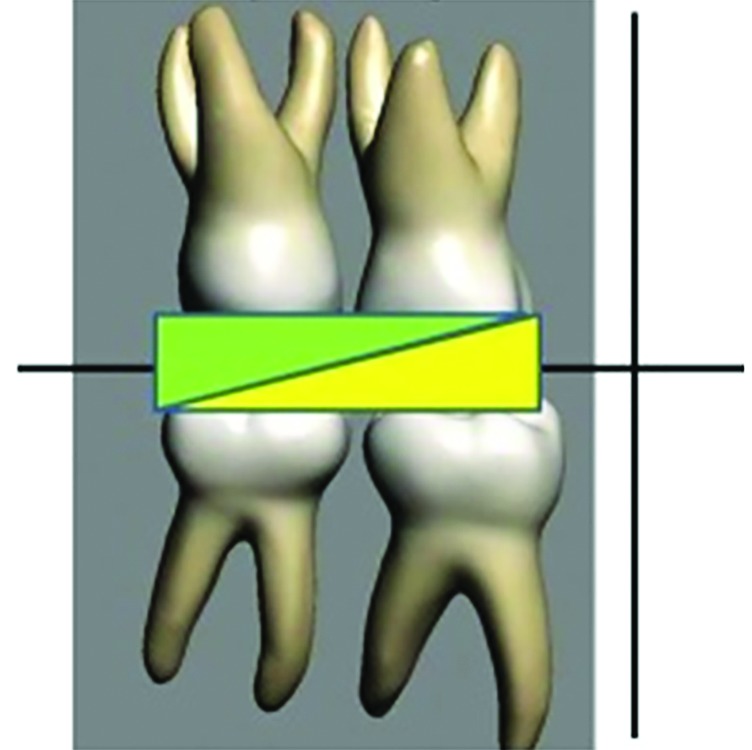
Graphical representation of slopes

- During the fabrication of the PDTs, a short modification of the tracks was considered by using self-cure acrylic resin in order to reduce a two-step laboratory procedure to a simple, single-step procedure, which is easy to fabricate and also economical.
- The design of the tracks was made as single track covering both the upper first and second deciduous molars and similarly in the lower arch, in order to reduce the complexity and precision involved in fabricating individual tracks.
- The design also involved a 20 to 45° slope in both the upper and lower tracks, where the distal slopes of the upper track meet the lower tack which is inclined mesially (Fig. 4).

**Fig. 5:**
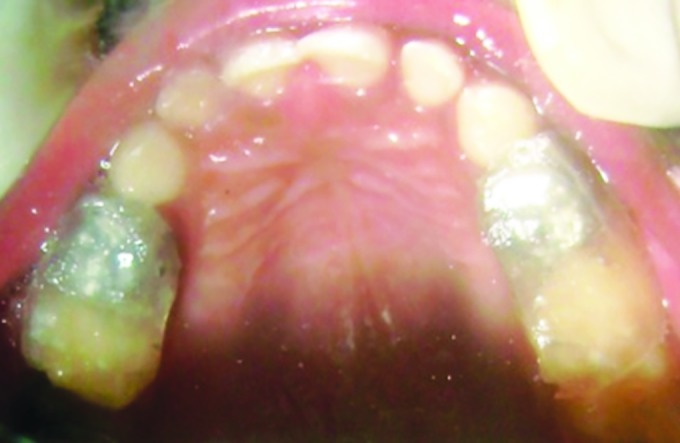
Modified PDTs in maxillary arch

**Fig. 6:**
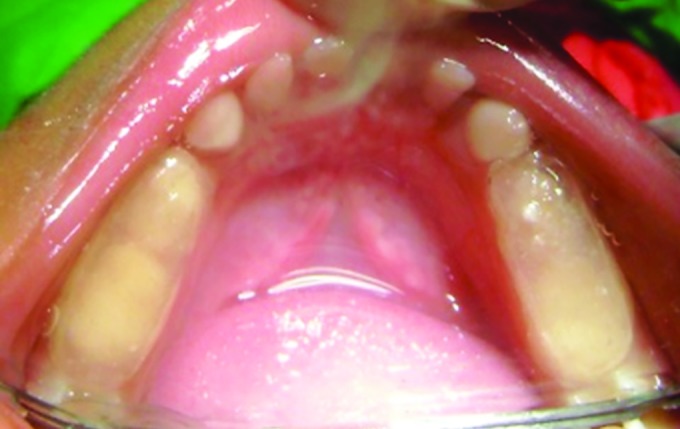
Modified PDTs in mandibular arch

**Fig. 7:**
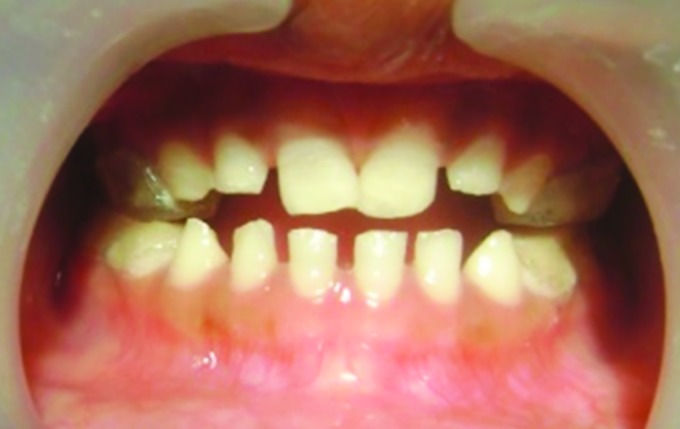
At occlusion

Once the curing is completed, excess material was trimmed and tracks were polished and cemented on the deciduous molars using type I glass ionomer cement (Figs 5 to 7). The whole procedure was done in single appointment and child cooperation was excellent.

The child was advised to have a soft diet for 2 to 3 days so that child gets adjusted to the appliance and advised to report back to us if there is any fractures or discomfort seen. Child was recalled for her next appointment after a month and the correction of crossbite was checked.

**Figs 8A to C:**
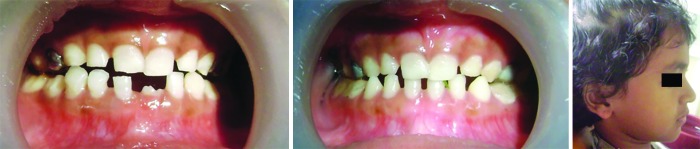
(A) After 2 months of treatment; (B) After 6 months of treatment; and (C) Lateral profile after treatment

After 2 months the crossbite was corrected (Fig. 8A). But the PDTs were kept in the oral cavity as there was possible relapse due to the forward closure of the mandible as suggested in previous studies.^9^ The child was recalled every month for the checkup and crossbites were corrected completely after a period of 6 months duration (Fig. 8B) and patient had convex profile (Fig. 8C). But the child was unable to maintain the oral hygiene properly and child had pain and bleeding on probing due to gingivitis in relation to upper deciduous molars.

**Figs 9A to D:**
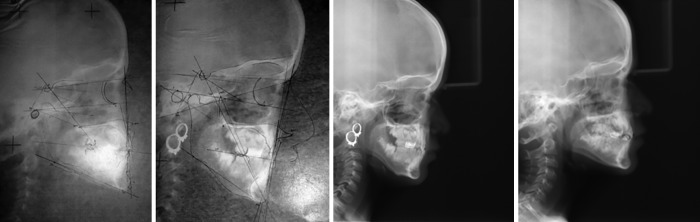
(A) Pretreatment cephalometric analysis; (B) posttreatment cephalometric analysis; (C) pretreatment radiograph; and (D) posttreatment radiograph

A cephalometric analysis was made before and after treatment to check the changes of the growth. It showed changes in the growth in normal ranges but there was definite change in the correction of crossbite and orientation of incisors (Fig. 9). Considering the correction of crossbite is adequate and chances of relapse are minute, the PDTs were removed and hand scaling was done and oral hygiene instructions were given, and it was instructed to mother to monitor the child’s oral hygiene regularly. Child was kept under observation with periodic recall for proper establishment of occlusion.

## DISCUSSION

For normal growth and development of the jaws, treating a crossbite at an earliest is to be considered.^7^ The ability to diagnose and treat the crossbites at the earliest depends on the knowledge of the practitioner about the features and consequences of the crossbite in a primary dentition.

Over the years, many treatment options were considered based on the clinical condition, duration of treatment required, patient and parent cooperation, easier fabrication, and economical appliances. The PDTs are one of the approaches for treating both anterior and posterior crossbite of primary dentition.^9^ In this case report, we are reporting a more simple, clinical, and laboratory technique, with less time and economical method of constructing PDTs and correcting the anterior crossbite efficiently and faster by also incorporating an inclined plane.

At centric occlusion the condyles are located concentrically in the glenoid fossae. Crossbites do not permit this and there will be muscular imbalance and temporo-mandibular joint (TMJ) displacement and abnormalities. But the studies have not found influence of TMJ disorders because of crossbite.^10^

Planas direct tracks work by repositioning the mandible, and by modifying the TMJ apparatus. That is, when PDTs are in place, the condyles are positioned in the most posterior position. Due to the ongoing growth, the components in the TMJ apparatus like glenoid fossae, ligaments, and joint spaces accommodate and modify to the new position of the growing condyles. This prevents the establishment of morphological and positional asymmetries in young children and allowing a more symmetrical craniofacial development.^7^

Hence, in this case, the PDTs were used to raise the bite and place an incline of 20 to 45° to occlusal plane, which brought the condyles into the most posterior position, without causing any damage to the growth. This was the basic principle used in Halls technique^11^ or twin-block appliances.^12^

In these cases, we have used an easily available, economic, biocomfortable material with good strength (self-cure acrylic resin) instead of composite resin for the fabrication of the PDTs and the delivery of the appliance was done in a single appointment, which reduces the laboratory procedure, appointment time, and increases patient cooperation, and is important as the child concentration and attention is short in preschool children.^7^

The cementation of the PDTs was done using glass ionomer luting, which does not bond with the self-cure resin but retained due to mechanical bonding. The chances of fracture of the PDTs are high because the self-cure resin is not as strong as composites and the occlusal load by the deciduous teeth is lesser than permanent tooth.^11^ Fracture of the PDTs was not observed in this case, but there was debonding of the PDTs of the upper arch, which was cemented immediately.

In this case, the correction of the anterior crossbite was edge-to-edge relation by the end of 1 month, and full correction was achieved in a span of 3 months time. The appliance was retained for 6 months, to stop any possibility of relapse and then removed, which was in accordance with the previous studies.^9^

Cephalometric analysis was done in this case, for the evaluation of growth changes seen in the child before and after the treatment and also the effect of the PDTs on the normal growth of the child (Table 1). Evaluation showed that PDTs did not affect the normal growth and development of the child and rather modify it and provides the favorable growth in the anteroposterior direction as the obstruction for existing growth potential is cleared and continuum of growth leads to possible adjustment.^13^

**Table 1:** **Table 1:** Cephalometric reading before and after treatment

*Descriptor*		*Patient data before treatment*		*Patient data after treatment*	
*Skeletal*					
Facial angle		90		88	
Angle convexity		–4		–1	
A-B plane		–3		–4.5	
Mandibular plane		23		27	
Y-axis		52		56	
*Dental*					
Occlusal plane		5		11	
Upper incisor to lower incisor		171		151	
Lower incisor to occlusal plane		81		81	
Lower incisor to mand		68		84	
84 upper incisor to A-Pog		3.0		4	

With this appliance, good results can be obtained by correcting crossbites in primary dentition. But the success of this appliance in children with skeletal discrepancy is not been established yet. There may be a requirement of second phase of treatment when the permanent dentition erupts, which might be of minimal duration.
